# The Co-Chaperone HspBP1 Is a Novel Component of Stress Granules that Regulates Their Formation

**DOI:** 10.3390/cells9040825

**Published:** 2020-03-29

**Authors:** Hicham Mahboubi, Ossama Moujaber, Mohamed Kodiha, Ursula Stochaj

**Affiliations:** Department of Physiology McGill University, 3655 Promenade Sir William Osler, Montreal, PQ H3G 1Y6, Canada

**Keywords:** HspBP1, stress response, stress granule, chaperone, co-chaperone, proteostasis

## Abstract

The co-chaperone HspBP1 interacts with members of the hsp70 family, but also provides chaperone-independent functions. We report here novel biological properties of HspBP1 that are relevant to the formation of cytoplasmic stress granules (SGs). SG assembly is a conserved reaction to environmental or pathological insults and part of the cellular stress response. Our study reveals that HspBP1 (1) is an integral SG constituent, and (2) a regulator of SG assembly. Oxidative stress relocates HspBP1 to SGs, where it co-localizes with granule marker proteins and polyA-RNA. Mass spectrometry and co-immunoprecipitation identified novel HspBP1-binding partners that are critical for SG biology. Specifically, HspBP1 associates with the SG proteins G3BP1, HuR and TIA-1/TIAR. HspBP1 also interacts with polyA-RNA in vivo and binds directly RNA homopolymers in vitro. Multiple lines of evidence and single-granule analyses demonstrate that HspBP1 is crucial for SG biogenesis. Thus, HspBP1 knockdown interferes with stress-induced SG assembly. By contrast, HspBP1 overexpression promotes SG formation in the absence of stress. Notably, the hsp70-binding domains of HspBP1 regulate SG production in unstressed cells. Taken together, we identified novel HspBP1 activities that control SG formation. These features expand HspBP1’s role in the cellular stress response and provide new mechanistic insights into SG biogenesis.

## 1. Introduction

Molecular chaperones and their binding partners regulate cellular proteostasis under all growth conditions. During stress, the activities of heat shock proteins are indispensable [1,2,3]. In particular, members of the hsp70 family and their co-chaperones promote stress survival and stimulate the recovery from cellular insults [2]. The proteostasis network is comprised of a large number of chaperones and their co-factors. This network is dynamic and adjusts to changes in cell physiology [4,5]. Hsp70 family members are pillars of the chaperone network and protect cells against proteotoxic insults [6]. 

The chaperone functions of hsp70s are regulated by a diverse group of interacting proteins, collectively called co-chaperones. One of these co-chaperones, HspBP1 (hsp70-binding protein 1), serves as a nucleotide exchange factor for hsp70 and inhibits the co-chaperone CHIP. These HspBP1 functions regulate cellular proteostasis and are relevant to human health [7,8,9]. For example, through inhibition of hsp70 HspBP1 is believed to facilitate drug-induced apoptosis in cultured cervical and gastric adenocarcinoma cells [10]. Notably, HspBP1 concentrations are often increased in tumor biopsies and some cancer cells [10,11,12,13]. For example, high *HSPBP1* gene expression or protein abundance has been observed for glioma, neuroblastoma, as well as hepatocellular, prostate and lung carcinoma cells. The high levels of HspBP1 in neurons may contribute to the development of neurodegenerative diseases [9], while secreted HspBP1 could modulate the extracellular activities of hsp70s [14]. Together, these studies indicate that HspBP1 participates in a wide variety of cell, tissue and organ functions, both under physiological and pathophysiological conditions.

Purified HspBP1 and hsc70 interact [8], but their subcellular localization is not coordinated in stressed cells [15,16]. This may suggest that HspBP1 engages in cellular activities that do not rely on hsc70 or other members of the chaperone family. Indeed, the role of HspBP1 in transcription supports this idea [17]. At present, the contributions of HspBP1 to cell homeostasis are not fully understood. This applies especially to conditions that elicit cellular stress. 

Cytoplasmic stress granules (SGs) are produced when mRNA translation is restricted. SGs are non-membrane-bound granular assemblies that contain different RNAs, RNA-binding proteins, components of the small ribosomal subunit and signaling molecules [18,19,20,21]. SG biogenesis occurs by phase separation ([22] and references therein); it is a conserved eukaryotic response to oxidants and other stressors [21,23]. SGs regulate mRNA stability, translation, and cell fate [20,24,25]. They are also key players for human health, since SG-like granules are implicated in cancer, neurodegenerative disorders and virus infections [19,23,26,27,28,29]. Notably, SG assembly is compromised in senescent cells, which impairs the stress response in aging organisms ([30] and references therein). Several proteins have a well-defined role for SG assembly. As such, G3BP1 and TIA-1/TIAR are critical to nucleate SGs during stress. Notably, their overexpression promotes SG formation, even in the absence of cellular insults [31,32,33]. Chaperones and co-chaperones, such as hsp70, also control granulostasis [34,35,36,37,38,39,40]. 

Eukaryotic cells produce a variety of RNA granules in the cytoplasm that modulate cellular homeostasis (reviewed in [30]). Aside from SGs, processing bodies (PBs) also regulate the fate of mRNA. These cytoplasmic compartments are present in unstressed and stressed cells, where they participate in mRNA decapping and degradation [41,42]. Although there is partial overlap in the composition of PBs and SGs [43], some proteins show a granule specific distribution. For example, the mRNA-binding proteins G3BP1, TIA-1/TIAR and HuR provide SG markers [31,32,44], whereas the decapping protein Dcp1 demarcates PBs [45]. 

The importance of chaperone networks for cellular homeostasis under normal, stress and pathological conditions is well-established [46]. HspBP1 is part of this network, but its contributions to proteostasis are not fully defined. The current study begins to fill these knowledge gaps. Specifically, we provide mechanistic insights that demonstrate the crucial role of HspBP1 for SG formation. Furthermore, we identify HspBP1 as an RNA-binding protein in vitro and in vivo. Our research places HspBP1 at the junction of proteostasis and RNA homeostasis. This new information is relevant to the physiology of healthy cells, aging, and a large spectrum of human diseases [30].

## 2. Materials and Methods

### 2.1. Cell Culture and Exposure to Stress

The following cell lines were used: HeLa (human cervix carcinoma; from J. White, McGill University), OK96T (opossum kidney; J. Orlowski, McGill University) and NIH-3T3 (mouse fibroblast; I. Gallouzi, McGill University). All cell lines were purchased originally from ATCC (American Type Culture Collection). The different cell lines used in this study were grown on poly-lysine coated cover slips. Oxidative stress was induced by incubation with vehicle (ethanol) or 2 mM diethyl maleate (DEM) for 4 hours at 37 °C, unless stated otherwise. The detailed treatment protocol has been published by us [47].

### 2.2. Antibodies and Pharmacological Reagents

Primary antibodies against HspBP1 (sc-34256), eIF2α (sc-30882), TIA-1/TIAR (sc-28237), HuR (sc-5261), Dcp1 (sc-100706) and hsp90 (sc-515081) were purchased from Santa Cruz Biotechnology, antibodies against hsp72 (SPA-812), hsc70/hsp72 (SPA-822), and Grp94 (SPA-850) were from Enzo, anti-G3BP1 (clone 23/G3BP1) from BD Transduction Laboratories, anti-phospho-eIF2α (p-Ser51; #3597) from Cell Signaling, and antibodies against actin from Chemicon (mab1501). Fluorescently labeled and HRP-coupled secondary antibodies were from Jackson ImmunoResearch. The secondary antibodies were affinity-purified and cross-adsorbed against antibodies from multiple other species. For immunofluorescence, all secondary antibodies were generated in donkeys. Apoptozole (Calbiochem) was dissolved in DMSO. For the experiments in Figure 9, the final concentration was 10 µM apoptozole and 0.2% DMSO in growth medium.

### 2.3. Immunofluorescence and Microscopy

Protocols for immunostaining have been described previously [47]. Nuclei were visualized with 4′,6-diamidino-2-phenylindole (DAPI). Images were acquired using the multi-track mode with a Zeiss LSM510 or LSM780. Appropriate filter settings were chosen to minimize cross-talk between the channels. Images were processed in Photoshop 8.0. 

### 2.4. SG Quantification and Statistical Analyses

Image acquisition for SG quantification followed published procedures [48]; HuR, G3BP1 or TIA served as markers to identify SGs. The number and area of SGs was quantified in at least 20 cells for each condition with MetaXpress software (Molecular Devices). The average SG number/cell and average SG area/cell were calculated for each data point. One-Way ANOVA combined with Bonferroni correction was applied for multiple comparison tests. A two-tailed Student’s t-test was used to identify statistically significant differences between two groups. Untreated control samples served as reference for statistical evaluations.

### 2.5. Protein Crosslinking and Indirect Immunoprecipitation

Reversible protein crosslinking with Dithiobis(succinimidylpropionate) (DSP) and immunoprecipita tion essentially followed published procedures [49], with the following modifications: crosslinking was carried out for 15 min at 37 °C; samples were then rinsed with PBS and 50 mM Tris HCl pH 7.4 containing 150 mM NaCl and stored at −70 °C. Under these conditions, nonspecific precipitation of unrelated proteins is low [49].

### 2.6. Mass Spectrometry

HeLa cells were grown under control conditions or exposed to DEM, and proteins were reversibly cross-linked with DSP. Protein complexes were isolated by immunoprecipitation with antibodies against HspBP1. Proteins were separated in a 7.5% to 15% SDS gel and visualized with GelCode Blue Stain (Pierce), following the company’s protocol. Stained bands were excised from the gel, reduced (10 mM DTT, 10 min), alkylated (55 mM iodoacetamide, 30 min), and digested overnight with trypsin (12 ng/μL) [50]. The resulting peptide digests were subjected to reverse phase separation, followed by MSMS on a Bruker HCT Ultra ion trap mass spectrometer. Data files were formatted to mgf files with Bruker ‘Compass Data Analysis’ software (standard settings) and searched on the *Homo sapiens* NCBI database (version NCBInr 2008), using Mascot v. 2.2 (Matrix Sciences). Protein identifications are based on unambiguous peptides with a Mowse score better than 47 (random probability value *p* < 0.05). Several peptides derived from G3BP1 were identified with this approach. Each peptide passed the confidence test, with a cutoff set at *p* < 0.05.

### 2.7. Oligo(dT) Binding Assay

PolyA-RNA protein complexes were purified using an oligo(dT)-binding assay previously described [47]. The specificity of this binding assay was also demonstrated earlier [47]. 

### 2.8. In Vitro RNA Homopolymer Binding Assay

The assay was carried out as published [47], with the following modifications. For the initial step, purified HspBP1 protein was incubated with homopolymer-coupled resins for 10 min at room temperature. The first wash included 2 mg/ml heparin; the second wash was performed with 1 M NaCl. Bound protein was eluted in gel sample buffer (15 min at 95 °C) and examined by Western blotting with antibodies against HspBP1.

### 2.9. Western Blotting

Methods for Western blotting and quantification of ECL signals have been described [49,51].

### 2.10. In Situ Hybridization of PolyA-RNA

PolyA-RNA was localized by hybridization with Cy3-labeled oligo-dT(50) [48,52]. In brief, control and DEM-treated cells were fixed for 15 min with 3.7% formaldehyde at room temperature. Following permeabilization with 0.3% Triton-X100 in PBS (room temperature), samples were rinsed with PBS and incubated with pre-hybridization buffer (2Χ SSC, 20% formamide, 2 mg/ml BSA, 1 mg/ml yeast tRNA; 15 min, 37 °C). The subsequent hybridization was carried out in pre-hybridization buffer supplemented with 10% dextran sulfate and 1 nmol/ml Cy3-oligo-dT(50) (overnight, 37 °C). After washing with 2Χ SSC, 20% formamide (5 min, 42 °C), 2Χ SSC (5 min, 42 °C), 1Χ SSC (5 min, room temperature), and PBS (5 min, room temperature), samples were stained with DAPI and mounted in Vectashield.

### 2.11. Transient Transfection

Cells were transiently transfected with different plasmids using Lipofectamine 2000 (Invitrogen) essentially according to the manufacturer’s instruction. Plasmids encoding scrambled or shRNA were obtained from SA Biosciences (Frederick, MD). For knockdown experiments, transfected cells were analyzed 4 days after transfection. In overexpression studies, cells were examined one day after transfection.

## 3. Results and Discussion

### 3.1. HspBP1 Is a Component of Oxidant-Induced SGs, but Does Not Concentrate in Cytoplasmic Processing Bodies

**Figure 1:**
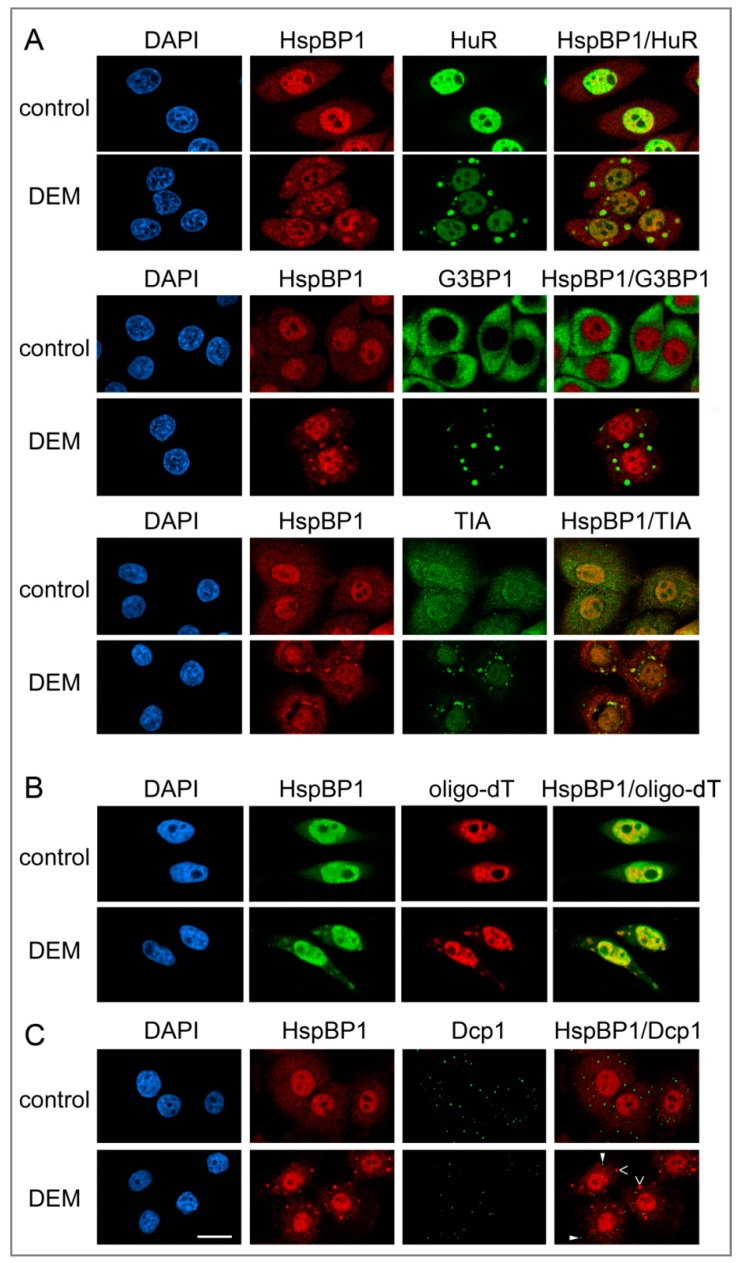
In response to oxidative stress, HspBP1 concentrates in cytoplasmic SGs, but not in PBs. HeLa cells were incubated for 4 hours with vehicle (control) or DEM, fixed and processed for indirect immunofluorescence and in situ hybridization (Materials and Methods). Nuclei were demarcated with DAPI. (**A**) HspBP1 located together with the SG markers HuR, G3BP1 or TIA. With the antibody used here, TIA was detected in both SGs and processing bodies (PBs). (**B**) HspBP1 co-localized with polyA-RNA in SGs. HeLa cells were treated 4 hours with vehicle or DEM. Samples were hybridized with Cy3-labeled oligo-dT(50) and subsequently immunostained with antibodies against HspBP1. (**C**) HspBP1 did not concentrate in PBs under control or oxidative stress conditions. Co-immunostaining was performed with antibodies against HspBP1 and the PB marker Dcp1. Some SGs (>) and PBs (►) are marked. Scale bar is 20 μm.

Our study was designed to better define the role of HspBP1 in the mammalian stress response. A first set of experiments examined how oxidative stress affects the subcellular distribution of HspBP1 (Figure 1). To this end, we incubated HeLa cells with diethyl maleate (DEM). This compound elicits oxidative stress, stimulates eIF2α phosphorylation and promotes the formation of SGs that contain the marker proteins HuR, G3BP1 and TIA-1/TIAR (here referred to as TIA) [47,48,51,53]. DEM induces the assembly of canonical SGs, which requires eIF2α phosphorylation [25]. In addition to marker proteins, bona fide SGs contain polyA-RNA, which can be detected by in situ hybridization with fluorescently labeled oligo-dT(50) [48]. 

Under normal conditions, endogenous HspBP1 was present in the nucleus and cytoplasm. However, upon DEM treatment the co-chaperone concentrated in large cytoplasmic compartments that were reminiscent of SGs (Figure 1). To characterize these compartments, we performed double immunolabeling with different SG markers. Figure 1A shows that HspBP1 co-localized with HuR, G3BP1 and TIA, which are established SG components. As observed by others, a portion of TIA was also present in processing bodies (PBs, [54]). The oxidant-induced association of HspBP1 with SGs was not limited to HeLa cells; it also occurred in other cell lines that are derived from different tissues and species (Supplemental Figure S1A). Notably, DEM-SGs did not accumulate hsc70, a highly abundant member of the hsp70 family (Supplemental Figure S1B). The combination of in situ hybridization and immunostaining revealed that HspBP1 also co-localized with polyA-RNA in cytoplasmic granules (Figure 1B). 

Stress exposure frequently stimulates the synthesis of chaperones and their co-factors. Although heat shock has no profound effect on HspBP1 concentrations [15,16], DEM caused a small but significant rise in HspBP1 levels. Specifically, following oxidative stress, HspBP1 abundance increased to ~140% of controls (Supplemental Figure S2). Under the same conditions, no significant changes were observed for HuR, G3BP1, TIA or the chaperone hsc70 (Supplemental Figure S2). Stressors that trigger SG assembly often increase the phosphorylation of translation initiation factor eIF2α on serine 51 [37]. We have shown previously that DEM stimulates eIF2α modification [25]. Supplemental Figure S3A,B confirms our observation by indirect immunofluorescence and Western blotting. 

Processing bodies (PBs) are functionally linked to SGs. Several proteins involved in RNA metabolism reside in both types of granules. Furthermore, some forms of stress enhance PB assembly [55]. To test whether HspBP1 is present in PBs, cells were stained with antibodies against HspBP1 and Dcp1, a PB marker that does not accumulate in SGs. HspBP1 was not detected in PBs under normal or stress conditions (Figure 1C). This is consistent with results for TIA (Figure 1A). While TIA located to both SGs and PBs, HspBP1 was only present in SGs. 

Taken together, our results establish that HspBP1 is an authentic constituent of oxidant-induced SGs. The stress-dependent relocation of HspBP1 to SGs was conserved among mammalian cells and observed for different cell types. Moreover, HspBP1 associated with SGs, but not with PBs, suggesting granule-specific functions. Our results expand the repertoire of SG components to a co-chaperone that is present in a wide variety of cells. This is relevant, because the current knowledge on mammalian SG composition is incomplete, especially as it relates to chaperones and their co-factors [35,36,37,38,39,40]. Given that HspBP1 has co-chaperone activities, the present study identified a new link that connects SGs to the proteostasis network. 

### 3.2. HspBP1 Binds the SG Marker Proteins G3BP1, HuR and TIA

**Figure 2:**
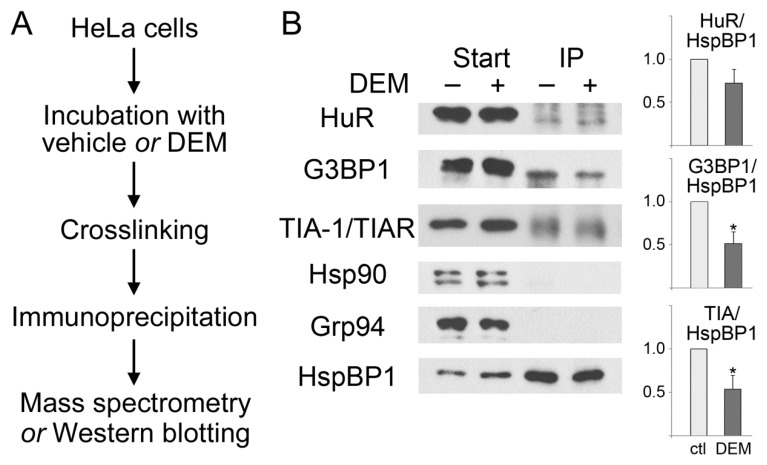
HspBP1 associates with SG proteins. Protein complexes were immunopurified with antibodies against HspBP1 from control and DEM-treated cells. (**A**) Workflow of the experiment. (**B**) Western blotting. Samples were incubated with vehicle (−) or DEM (+) as specified. Crude extracts (Start) and immunoprecipitates (IP) were analyzed with antibodies against HuR, G3BP1, TIA-1/TIAR, hsp90 or Grp94 as indicated. Filters were stripped and reprobed with antibodies against HspBP1. ECL signals were quantified to determine the stress-dependent changes in the association of HspBP1 with different binding partners. The ratio of interacting protein/HspBP1 was normalized to control conditions. Means +SEM are shown for three independent experiments. The student’s t-test identified significant differences; * *p* < 0.05. No binding of HspBP1 to hsp90 or Grp94 was detected under these conditions, demonstrating the specificity of our assay. For each protein, all lanes are from the same blot.

Mammalian SGs contain only a limited number of chaperones, including hsp70, hsp90, DNAJB6, and the small heat shock proteins HspB8/Hsp22 and Hsp27/Hsp25/HspB1 ([35,36,37,38,39,40], and references therein). It was therefore conceivable that HspBP1 associates with non-chaperone SG components. To identify such interacting proteins, we used control and stressed cells to crosslink and immunoprecipitate protein complexes that contained HspBP1. The overall strategy of the experiment is depicted in Figure 2A. Immunoprecipitates were subjected to mass spectrometry, and G3BP1 was identified as an HspBP1-associated factor. These results were verified independently by indirect immunoprecipitation combined with Western blotting (Figure 2B). HspBP1 co-purified with G3BP1, both under normal and oxidative stress conditions. Similar results were obtained for TIA and HuR. All of these interactions were detected in control and stressed cells. Interestingly, the associations were diminished by stress (Figure 2B). This indicates that the interaction of HspBP1 with G3BP1, TIA and HuR also plays a role under non-stress conditions. The physiological significance will have to be determined in future studies. It should be emphasized that the connection between HspBP1 and SG components was specific, because HspBP1 did not co-purify with hsp90 or Grp94 in control or DEM-treated cells (Figure 2B).

Collectively, our data demonstrate that HspBP1 formed complexes with several mRNA-binding proteins. These proteins included SG nucleators as well as regulators of mRNA stability and translation. The results are consistent with the model that HspBP1 plays a role for granulostasis and RNA homeostasis. We further explored our hypotheses with the experiments discussed in the following sections.

### 3.3. HspBP1 Association with SGs during Granule Formation and Recovery

**Figure 3:**
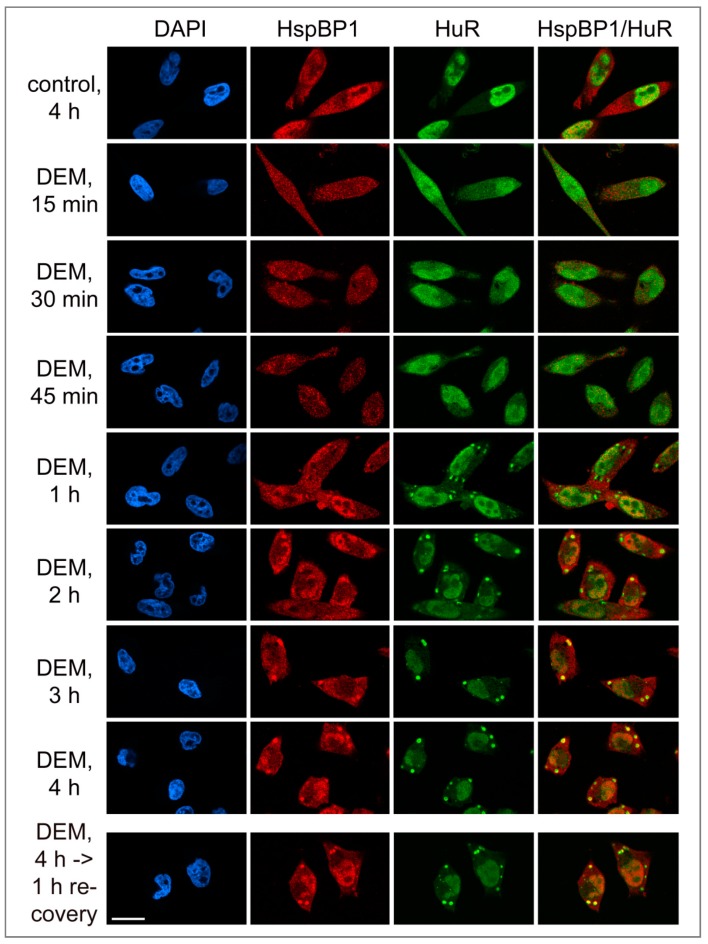
Kinetics of HspBP1 recruitment to stress granules. HeLa cells were treated with vehicle (control) or exposed to DEM. After a 4-hour DEM treatment, cells were transferred to medium without DEM for recovery (bottom panels). Samples were fixed at the time point indicated, and the distribution of HspBP1 and HuR was examined by immunocytochemistry (Materials and Methods). HspBP1 remained associated with SGs during the recovery from stress. DAPI demarcated nuclei; scale bar is 20 μm.

**Figure 4:**
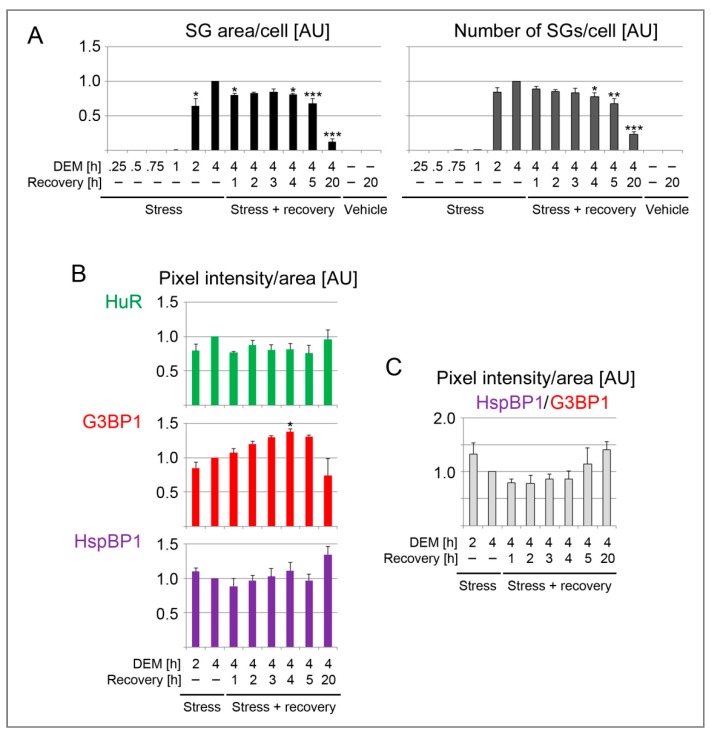
Quantification of SG parameters in DEM-treated HeLa cells. Each dataset was normalized to the 4-hour DEM treatment. (**A**) HeLa cells were incubated with growth medium containing 2 mM DEM or vehicle for the times indicated. After the 4-hour treatment, cells were transferred to growth medium for recovery. The results are shown as average +SEM for 3 to 4 independent experiments. For each experiment, at least 44 cells were analyzed for every data point. G3BP1 and HuR served as SG markers. One-Way ANOVA with Bonferroni correction was performed for 2-hour DEM, 4-hour DEM and all stress recovery points. The results for 4-hour DEM were used as reference. * *p* < 0.05, ** *p* < 0.01, *** *p* < 0.001. (**B**) The SG association of HuR, G3BP1 and HspBP1 was measured for individual SGs. Different time points were assessed during stress and recovery. The pixel intensity/SG area was quantified for 3 to 4 independent experiments. Each dataset was normalized to the 4-hour DEM treatment. Except for overnight recovery (317 SGs), the total number of SGs examined for each time point was between 1145 and 2059. * *p* < 0.05. (**C**) The ratio HspBP1/G3BP1 was calculated for the pixel intensity/area. Individual datasets were normalized to the results for 4-hour DEM incubation. AU, arbitrary units.

To better define the relationship between SGs and HspBP1, we measured several SG parameters at different time points during a 4-hour DEM exposure and upon stress recovery (Figure 3 and Figure 4). Figure 4A shows the average SG area/cell and the number of SGs/cell. All results were normalized to the 4-hour DEM incubation. No SGs were detected with the vehicle or after the recovery from vehicle incubation. 

Following a 1-hour DEM treatment, few SGs were present. These granules contained HuR and G3BP1, and they stained positive for HspBP1 (Figure 3 and Figure 4). After 2 hours, the granules became larger and more abundant. At the end of the 4-hour stress period, the SG area/cell and number of SGs/cell reached a maximum. Both features changed markedly when the stressor was removed. Specifically, the SG area/cell and the number of SGs/cell diminished significantly at 4 hours of recovery. These changes were more pronounced when the recovery period was extended to 20 hours. At this point, only few SGs were present. Throughout the recovery from DEM stress, HspBP1 was present in SGs (Figure 3 and Figure 4).

The quantitative image analyses depicted in Figure 4 focused on SG properties. Specifically, we quantified the granule association of HuR, G3BP1 and HspBP1 for individual SGs. Since only few granules were generated prior to 2 hours of stress exposure, we evaluated later time points and the subsequent recovery period. G3BP1 pixel intensities/area increased during stress exposure. Surprisingly, this value continued to rise when DEM was removed, peaking at 4 hours of recovery. By contrast, no significant changes were observed for HspBP1 and HuR (Figure 4B). Interestingly, after a 20-hour recovery period, the few granules that persisted were enriched for HspBP1, but depleted for G3BP1.

Since G3BP1 serves as a SG-nucleating protein, we evaluated its relationship to HspBP1 at different stages of SG formation and disassembly. The HspBP1/G3BP1 ratio (Figure 4C) shows that their relative abundance in granules differs at distinct stages of the SG cycle. Compared to G3BP1, HspBP1 was particularly abundant in newly formed (2 hours DEM) and persistent SGs (20 hours recovery). Together, our results are consistent with other studies that revealed the dynamic changes of SG composition during stress and recovery periods [23,56,57].

It should be noted that we used antibodies to evaluate SG characteristics. Although some epitopes may become less accessible in SGs, tags can alter the subcellular distribution and other properties of a fusion protein. This was indeed observed for HspBP1, as epitope tags affected its distribution (data not shown). Therefore, we conducted our work with polyclonal antibodies that recognized full length HspBP1 and the deletion mutants discussed in Section 3.7. 

In this study, we provide evidence that HspBP1 located to SGs during different stages of granule formation and disassembly. Furthermore, our data support the model that HspBP1 granule recruitment and release during recovery differ from the nucleator G3BP1. Interestingly, relative to G3BP1, granules were enriched for HspBP1 at early stages of SG formation and late during disassembly. Future studies will have to determine how HspBP1 modulates SG properties during granule nucleation, maturation and dissolution. 

### 3.4. HspBP1 Binds PolyA-RNA In Vivo and Homopolymers In Vitro

**Figure 5:**
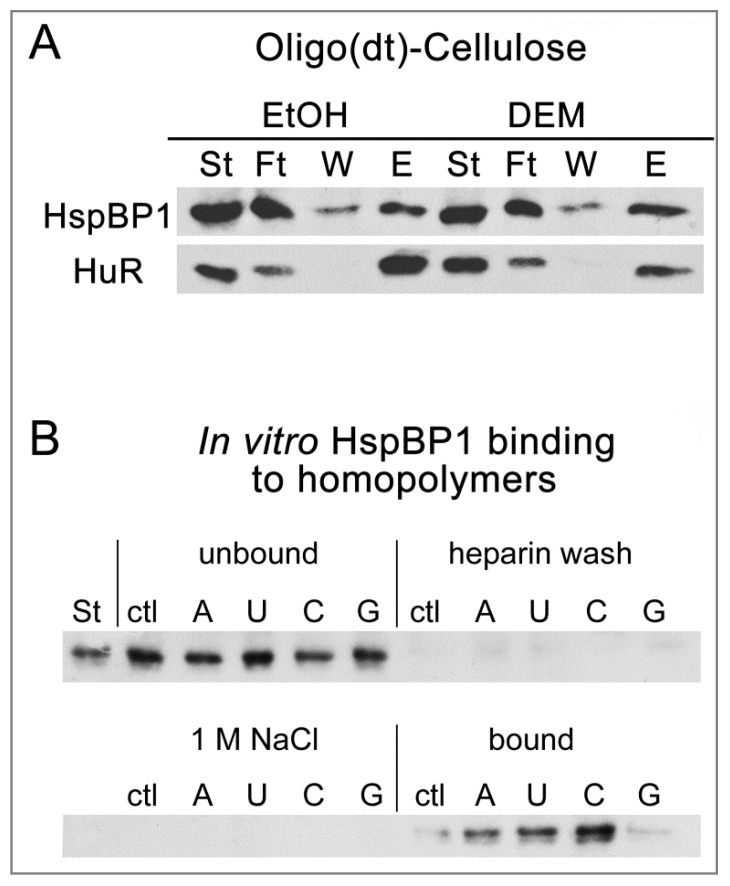
(**A**) HspBP1 interacts with RNA in vivo and in vitro. PolyA-RNA binding was evaluated as described in detail [47]. HeLa cells were incubated with vehicle (EtOH, ethanol) or 2mM DEM for 4 hours and extracts were prepared. Starting material (ST), flow through (Ft), wash (W) and material eluted from oligo(dt)-cellulose (E) was analyzed by Western blotting. HspBP1 associated with polyA-RNA under control (EtOH) and stress (DEM) conditions. HuR provided a positive control. (**B**) Purified HspBP1 binds RNA homopolymers in vitro. Control and homopolymer resins were incubated with purified HspBP1. Starting material (St), unbound, heparin and 1 M NaCl wash fractions, as well as bound material were examined by Western blotting with antibodies against HspBP1. All lanes for each filter were on the same blot.

SGs are enriched for RNA binding proteins, many of which contain glycine-rich domains [58]. Glycine residues are abundant in the N-terminal segment of HspBP1, and we showed here that HspBP1 is recruited to SGs (Figure 1 and Figure 3). This prompted us to examine whether HspBP1 associates with RNA. To this end, we purified polyA-RNA/protein complexes with oligo-(dT)-coupled cellulose from control and stressed cell (Figure 5A). The co-purification of HspBP1 was monitored by Western blotting, with HuR as a positive control. Figure 5A shows that a substantial portion of HspBP1 was recovered in the eluate (fraction E), both for normal and stress conditions. These interactions were specific, as we have demonstrated with previously published control experiments [47].

To determine whether HspBP1 interacts directly with RNA, an in vitro RNA homopolymer binding assay was performed [47,59]. Purified HspBP1 was incubated with polyA, polyU, polyC or polyG-coupled Sepharose (Figure 5B), with non-coupled Sepharose as a negative control. Beads were washed with heparin and 1M NaCl, and HspBP1 binding was evaluated. These experiments revealed that HspBP1 bound RNA directly, with preference for polyC, whereas no binding was observed for polyG. 

In summary, HspBP1 was part of polyA-RNA/protein complexes in growing cells. In vitro, HspBP1 associated with RNA in a sequence-specific fashion, and this binding was maintained under stringent conditions. Collectively, the interactions with RNA and SG proteins could stimulate HspBP1 recruitment to SGs. Our results suggest a unique role for HspBP1 in cellular physiology and stress responses in particular. Based on the associations with proteins and RNA we have identified here, HspBP1 may serve as a node that integrates the proteostasis network with regulators of RNA homeostasis. Given the preferential binding to polyC homopolymers in vitro, it will be interesting to determine whether HspBP1 and polyC-binding proteins participate in overlapping activities in vivo [60].

### 3.5. HspBP1 Knockdown Impairs SG Formation

**Figure 6:**
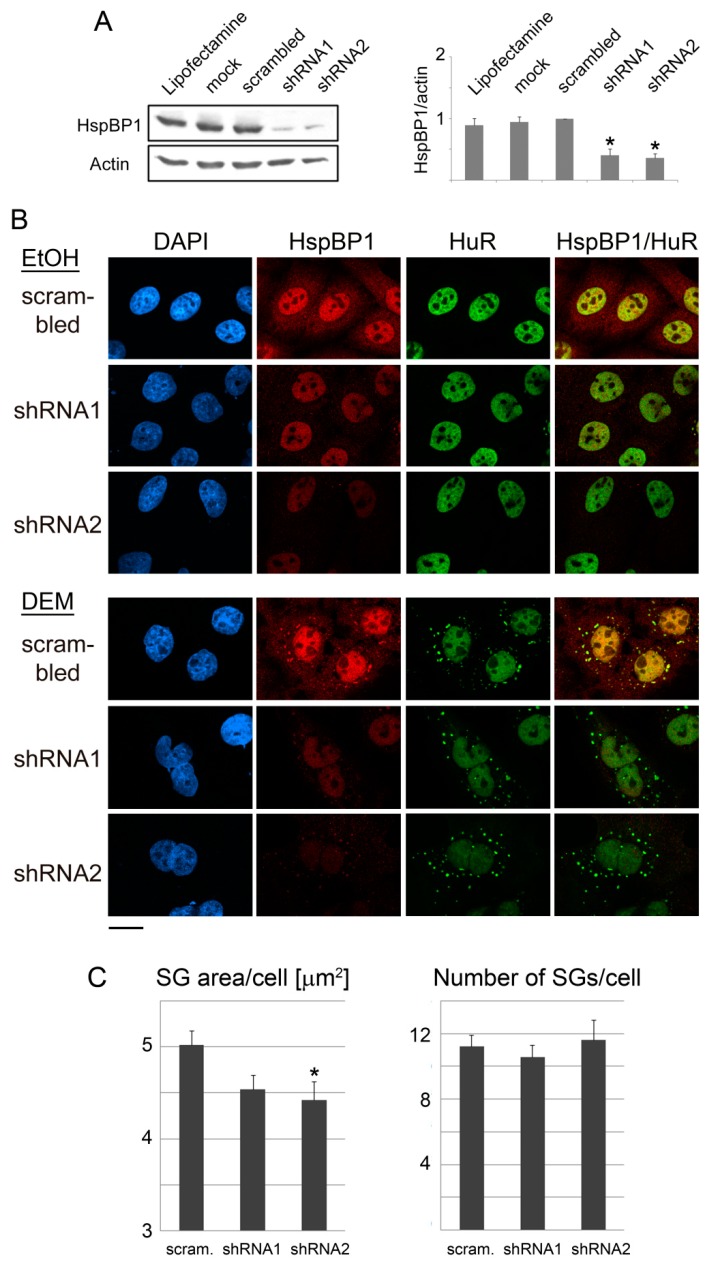
HspBP1 knockdown impairs SG formation. HeLa cells were incubated for 4 days with the transfection agent lipofectamine, mock-treated, transfected with a control plasmid (scrambled) or constructs targeting two different regions of the HspBP1 transcript (shRNA1 and shRNA2). (**A**) Crude cell extracts were probed for HspBP1; actin was used as loading control. Quantitative Western blotting revealed a significant depletion of HspBP1 for each of the sh-constructs. All lanes were on the same blot. Results are shown as average +SEM. Significant differences were identified with One-Way ANOVA combined with Bonferroni posthoc analysis; * *p* < 0.05. (**B**) HeLa cells were transfected with control DNA or individual HspBP1 knockdown plasmids and subsequently treated with DEM. SGs were identified with HuR. Scale bar is 20 μm. (**C**) Using HuR as a marker, the SG area/cell and number of SGs/cell were quantified for control and HspBP1 knockdown cells. Bar graphs depict averages +SEM. Significant differences were determined with One-Way ANOVA and Bonferroni correction, control cells served as the reference; scram., scrambled sequence. * *p* < 0.05.

A silencing strategy was employed to define the role of HspBP1 in SG formation. Control experiments showed that the transfection reagent, mock transfection, or a control plasmid had little effect on HspBP1 levels (Figure 6A). Plasmids shRNA1 and shRNA2 target two non-overlapping sequences of the HspBP1 mRNA, and both diminished significantly the HspBP1 protein abundance. We used these silencing constructs to examine the impact of HspBP1 knockdown on the formation of DEM-SGs. HspBP1 knockdown did not profoundly alter the kinetics of SG assembly, which was evaluated with the marker HuR. Granules appeared upon 2 hours of DEM treatment (Supplemental Figure S4), demonstrating that cells were still able to sense and respond to stress.

For an in-depth analysis, we quantified the number and size of SGs in knockdown cells. Notably, based on the marker HuR, SGs were considerably smaller upon HspBP1 depletion (Figure 6B). By contrast, the impact on granule numbers was more variable. The effects of HspBP1 knockdown were confirmed independently with two other granule markers, G3BP1 and TIA (Supplemental Figure S5). As seen with the marker HuR, HspBP1 depletion had little impact on the kinetics of oxidant-induced SG formation (not shown). This indicates that the major consequence of HspBP1 knockdown was impaired overall SG assembly, rather than delayed granule formation (Supplemental Figure S5). 

Taken together, our data suggest that HspBP1 determines SG properties, especially the size of granules. A reduction of HspBP1 abundance did not abolish SG formation. However, the size changes indicate significant impairment in granule biogenesis. Thus, we uncovered a novel and specific role for HspBP1 in granulostasis [34]. Our results also emphasize the importance of HspBP1 for the proper response to oxidative stress, a process controlled by the collaboration of proteostasis network components [61]. 

### 3.6. Full Length HspBP1 Overexpression Induces the Formation of Cytoplasmic SGs in the Absence of Stress 

**Figure 7:**
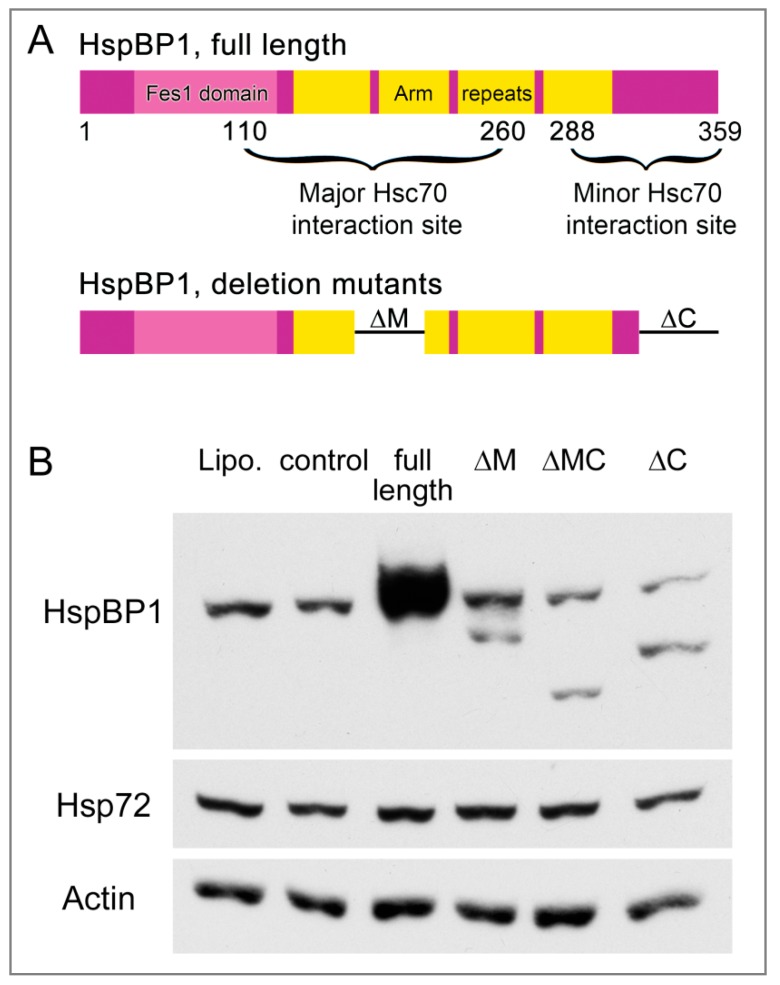
Production of HspBP1 full length protein and deletion mutants. (**A**) Organization of the HspBP1 protein. HspBP1 domains and the regions interacting with hsc70 are shown for the full length protein [16]. The organization of deletion mutants is also depicted. (**B**) Western blot analysis of full length and mutant HspBP1. Crude extracts were prepared for transfected HeLa cells and probed with antibodies against HspBP1 or hsp72. Actin was used as loading control. Lipo, lipofectamine; ctl, control plasmid; full length HspBP1, ΔM (deletion of residues 154–195), ΔMC (deletion of residues 154–195 and 314–359) ΔC (deletion of residues 314–359). All lanes were on the same blot.

**Figure 8:**
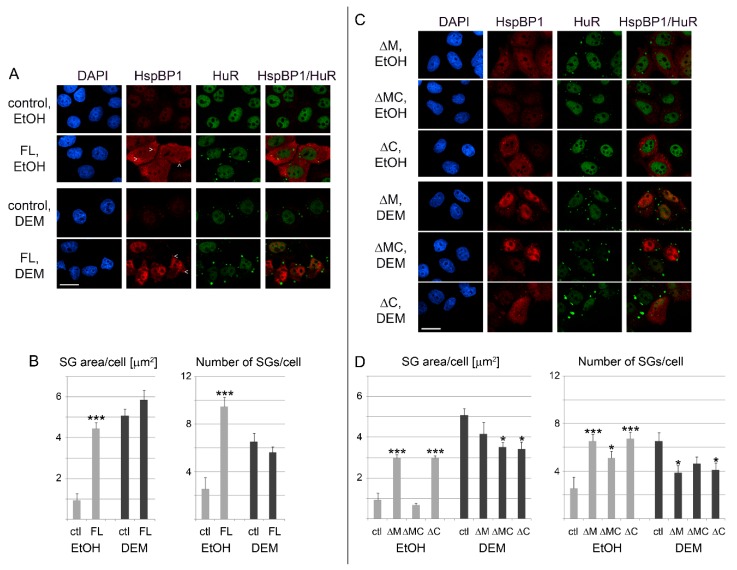
(**A**) HspBP1 overexpression induces the formation of SGs. HeLa cells were transiently transfected with plasmids encoding full length HspBP1 (FL). The SG marker HuR was used to monitor granule formation. All images were acquired with the same settings; signals for HspBP1 are therefore low in control cells. The position of several SGs is marked by arrowheads. Scale bar is 20 μm. (**B**) The effects of HspBP1 overexpression on SG area and number was quantified for a representative experiment. SG parameters were measured for 20 cells/condition, as described for Figure 6. Student’s t-test identified significant differences between controls and cells overexpressing *HSPBP1*. Comparisons were made within vehicle (EtOH) or DEM groups; *** *p* < 0.001. (**C**,**D**) Role of hsp70-interacting domains for SG production. The impact of HspBP1 deletion mutants on SG formation was analyzed for vehicle and DEM-treated cells as in part A and B. Statistical evaluation was performed with One-Way ANOVA plus Bonferroni correction, using control cells (ctl) as reference; * *p* < 0.05; *** *p* < 0.001. Scale bar is 20 μm.

Figure 6 shows that HspBP1 knockdown reduced SG size. This prompted us to examine the effects of HspBP1 overexpression. For initial studies, we fused a small epitope tag or fluorescent proteins to full length HspBP1. However, these fusion proteins failed to accumulate in DEM-SGs, suggesting that tagging prevented the proper targeting to granules (data not shown). Therefore, overexpression experiments were carried out with a non-tagged version of HspBP1. In transiently transfected cells, full length HspBP1 protein levels increased profoundly, as revealed by Western blotting and immunostaining (Figure 7 and Figure 8). Importantly, overexpression of full length HspBP1 led to SG formation, even in the absence of stress (Figure 8A).

Different mechanisms were uncovered for SG assembly when HspBP1 overexpression was compared with DEM treatment. While DEM-SGs rely on eIF2α phosphorylation [25], HspBP1 overexpression did not stimulate eIF2α phosphorylation. Two lines of evidence support this conclusion, Western blotting of crude cell extracts (Supplemental Figure S6A) and single-cell analysis (Supplemental Figure S6B). The results for HspBP1 are reminiscent to the overexpression of other granule-inducing proteins, such as G3BP1 and TIA [32,43]. These proteins promote SG-assembly without increasing the phosphorylation of eIF2α and in the absence of exogenous stress [23]. We conclude that HspBP1, like other polyA-RNA binding proteins, can induce granule formation when produced at high concentrations. Whether the relatively high abundance of HspBP1 in neurons [9] or cancer cells [10,11,12,13] stimulates RNA granule assembly is an exciting hypothesis that requires further exploration. 

### 3.7. The hsp70-Interaction Domains of HspBP1 Stimulate SG Assembly in the Absence of Stress 

HspBP1 serves as a co-chaperone for members of the hsp70 family [7,62] and can inhibit the hsp70 chaperone activity. Binding to the hsp70 ATPase domain is a prerequisite for this co-chaperone function. Major and minor interaction sites that include helices α5 and α17, respectively, associate with hsp70 (Figure 7A, [7]). The deletion of an internal region of HspBP1 that contains helix α5 reduces the binding to hsp70, and removal of both the major (residues 154 to 195; ΔM) and minor (residues 314 to 359; ΔC) interacting regions almost completely abolishes the HspBP1/hsp70 association [10]. 

To assess the contribution of hsp70-interacting sites to SG formation, we generated HspBP1 deletion mutants ΔM, ΔMC and ΔC. The mutant proteins are missing one or both of the hsp70-binding sites. We verified in transiently transfected cells that the mutant genes encode proteins of the expected molecular mass (Figure 7B). Supplemental Figure S7 confirms published observations [10] and verified that the association with hsp/hsc70s is reduced for these HspBP1 deletion mutants. In transiently transfected cells, we were unable to overexpress the mutant genes to the same extent as the wild type. Adjustment of DNA concentration or alternative transfection agents did not increase the levels of HspBP1 mutant proteins. This may reflect instability or toxicity of the deletion mutants. 

For the conditions selected here, the abundance of deletion mutants was similar to full length HspBP1. A comparison of the different proteins was therefore appropriate (Figure 8C,D). The most striking result was obtained for mutant ΔMC under non-stress conditions. Specifically, the SG area/cell was reduced profoundly for ΔMC when compared with ΔM and ΔC. At the same time, all mutant proteins produced a significantly higher number of SGs than control cells (Figure 8D, right panel). This indicates that the loss of both hsp70-interacting regions limits the total SG area per cell. 

We propose the following model for the results presented above. SGs are dynamic granules with continuous flux of macromolecules. Hsp70 inhibits the aggregation of SG-nucleating proteins; the chaperone also stimulates SG disassembly [31,35,57,63,64]. These processes are fine-tuned by HspBP1, which limits the impact of hsp70 on SGs. Consistent with this idea, the overexpression of full-length HspBP1, mutant ΔM or ΔC stimulated granule assembly without stress. Loss of both hsp70-interacting sites prevented HspBP1 from inhibiting hsp70. As a consequence, the mutant ΔMC failed to promote the formation of large SGs in unstressed cells. 

A different scenario emerged when HspBP1 was overexpressed during DEM-stress. Overexpression of full length HspBP1 caused significant changes in the SG area or number, whereas HspBP1 deletion mutants reduced both parameters (Figure 8B,D). Since mutant ΔMC does not bind hsp70s, our results indicate that yet to be defined HspBP1 interactors contribute to the proper SG assembly in stressed cells. Since we have shown that HspBP1 associates with non-chaperone SG components (Figure 2), it is tempting to speculate that these interactions also modulate granule characteristics. 

### 3.8. Effects of Pharmacological Inhibition of hsp70 on SGs

**Figure 9:**
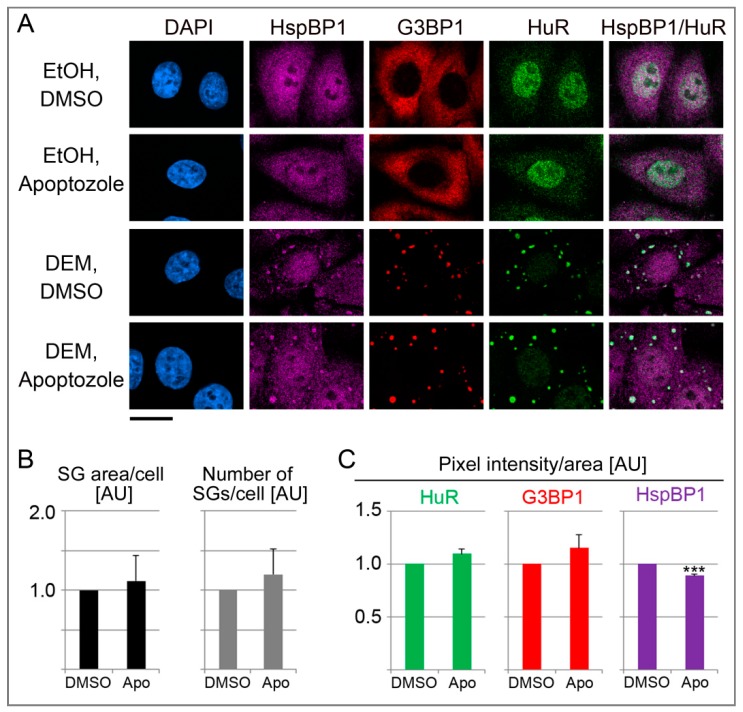
Hsp/hsc70 inhibition alters the properties of DEM-SGs. (**A**) HeLa cells were incubated with 2 mM DEM for 4 hours. The vehicle DMSO or 10 µM apoptozole were added together with ethanol (EtOH) or DEM. Scale bar is 20 μm. (**B**,**C**) Quantification is shown as average + SEM for two independent experiments. Results were normalized to vehicle controls. At least 54 cells or 355 SGs were evaluated for each experiment and data point. Significant differences were identified with Student’s *t*-test; *** *p* < 0.001. AU, arbitrary units.

The results depicted in Figure 8 suggest that HspBP1 controls SG properties, in part through its co-chaperone function. To examine this possibility further, we inhibited the hsp/hsc70 chaperone activity with apoptozole. This pharmacological compound binds the N-terminal ATPase domain of hsp70 family members [65]. When added together with DEM, the effects of apoptozole were variable, but overall, the compound increased the SG area/cell and the number of SGs/cell (Figure 9A,B). This observation further supports the hypothesis that the inhibition of hsp70 family members regulated SG formation. Such modulation can be achieved through co-chaperones like HspBP1, or pharmacological compounds. At the single SG level, apoptozole somewhat stimulated the association of HuR and G3BP1 with granules, whereas the abundance of HspBP1 in individual SGs was diminished (Figure 9C). Our analyses of single SGs substantiates that chaperones have a surveillance function for SGs [57]. Together with the experiments discussed in previous sections, our results with pharmacological inhibitors further validate the contribution of molecular chaperones to granulostasis.

## 4. Conclusions 

**Figure 10:**
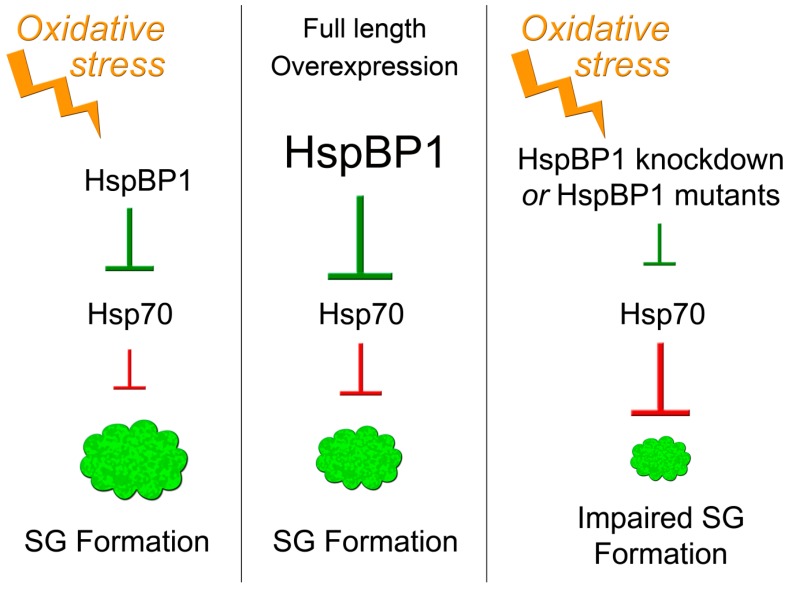
Simplified model depicting the role of HspBP1/hsp70 interactions for SG formation. Stress, such as the oxidant DEM, induces SG assembly. HspBP1, through its inhibition of hsp70, controls SG formation. Overexpression of HspBP1 triggers SG assembly, even in the absence of stress. HspBP1 knockdown or HspBP1 mutants with compromised hsp70 interaction impair SG assembly during oxidative stress. See text for details.

Chaperones and their binding partners are essential for proteostasis. Moreover, they determine cell survival during stress and disease [3]. Here, we examined the role of the co-chaperone HspBP1 for cells experiencing oxidative stress. Our study established HspBP1 as a novel component of oxidant-induced SGs and defined HspBP1 as a regulator of granule biogenesis (summarized in Figure 10). Here, we identified new binding partners for HspBP1, including the SG-nucleating proteins G3BP1 and TIA as well as the SG-resident HuR. Importantly, the potential of HspBP1 to interact with granule components is not limited to proteins. Our work uncovered for the first time, that HspBP1 associates with polyA-RNA in vivo and binds specific RNA sequences in vitro. We propose that the combination of RNA- and SG protein-binding facilitates the recruitment of HspBP1 to SGs. 

HspBP1 is not only an authentic SG component, it is also critical for proper granule formation. Our genetic approaches and mutant analyses support this conclusion. First, *HSPBP1* knockdown revealed that HspBP1 controls SG assembly in stressed cells. Second, *HSPBP1* overexpression induced SG assembly in the absence of stress or increased eIF2α phosphorylation. Evaluation of HspBP1 mutants suggests that the interaction HspBP1/hsp70 regulates granule size under non-stress conditions. 

Interestingly, in the context of stress, all HspBP1 mutants reduced the number of SGs/cell. This indicates that HspBP1 has chaperone-independent functions and is in line with recent studies [16,17]. Taken together, our research supports the idea that HspBP1 regulates granule production through multiple pathways; these pathways are dependent and independent of hsp70s. 

Our conclusions are supported by published models for SG assembly. It was proposed that protein chaperones contribute to granule remodeling during assembly and stress recovery [31,35,38,57,63,64]. Previous work examined the role of hsp70 for SG disassembly and granule quality control [35,38,57,63]. These studies focused in particular on hsp70, the CCT complex (chaperonin containing TCP1 ring complex), small heat shock protein hspB8, and the co-factors hsp40 and Bag3. 

Whereas earlier research evaluated predominantly the impact of the chaperone network on SG disassembly and granulostasis, the links between chaperones and SG formation are less well characterized. One exception is the CCT complex, which inhibits SG formation [38]. Our work goes beyond these observations, as we identified HspBP1 as a novel modulator of SG formation. While the HspBP1/hsp70 association is clearly important for granulostasis, we expect that HspBP1 has a broader role. For example, it is tempting to speculate that the glycine-rich N-terminal segment of HspBP1 contributes to SG assembly by promoting phase separation. Glycine-rich domains have been identified in a diverse group of proteins that promote liquid-liquid phase separation, including proteins located in PBs, the miRISC complex, and at the nuclear pore complex [66,67,68]. It is interesting to note that non-polysomal mRNA is required to generate protein aggregates during hsp70 inhibition [69]. Based on our study, HspBP1 is an ideal candidate to participate in these activities: the co-chaperone associates with polyA-RNA in growing cells and modulates hsp70’s chaperone activities.

In summary, the current study identified HspBP1 as an integral component of SGs and revealed its role in SG formation. Here, we presented new insights that implicate HspBP1 in multiple aspects of cellular homeostasis. In this scenario, HspBP1 connects components of the proteostasis network [61] with cellular RNA homeostasis. Given the importance of RNA granules and granulostasis for human health, our study sets the stage for future work to uncover the impact of HspBP1 on aging and a wide range of human diseases and disorders. 

## Supplementary Materials

The following are available online at https://www.mdpi.com/2073-4409/9/4/825/s1, Figure S1: (A) HspBP1 associates with stress granules in different mammalian cells. (B) Hsc70 does not concentrate in DEM- SGs. Figure S2: DEM increases the abundance of HspBP1. Figure S3: DEM increases the phosphorylation of eIF2α. Figure S4: Effect of HspBP1 depletion on the kinetics of SG formation. Figure S5: Quantification of granule parameters with G3BP1 and TIA as SG markers. Figure S6: HspBP1 overexpression does not enhance the phosphorylation of eIF2α. Figure S7: HspBP1 deletion mutants show diminished binding to hsp/hsc70s.
