# Supplementary material for: The Co-Chaperone HspBP1 Is a Novel Component of Stress Granules that Regulates Their Formation
Source: Cells. 2020 Mar 29;9(4):825. doi: 10.3390/cells9040825 (PMC7226807; doi:10.3390/cells9040825)
Supplement: Supplementary file 1 [file cells-09-00825-s001.pdf]

Supplementary Material

The co-chaperone HspBP1 is a novel component of stress granules that regulates their formation

Hicham Mahboubi, Ossama Moujaber, Mohamed Kodiha and Ursula Stochaj

Figure S1.

(**A**) **HspBP1 associates with stress granules in different mammalian cells.**

(**B**) **Hsc70 does not concentrate in DEM-SGs.**

Figure S2.

DEM increases the abundance of HspBP1.

Figure S3.

DEM increases the phosphorylation of eIF2α.

Figure S4.

Effect of HspBP1 depletion on the kinetics of SG formation.

Figure S5.

Quantification of granule parameters with G3BP1 and TIA as SG markers.

Figure S6:

HspBP1 overexpression does not enhance the phosphorylation of eIF2α.

Figure S7:

HspBP1 deletion mutants show diminished binding to hsp/hsc70s.

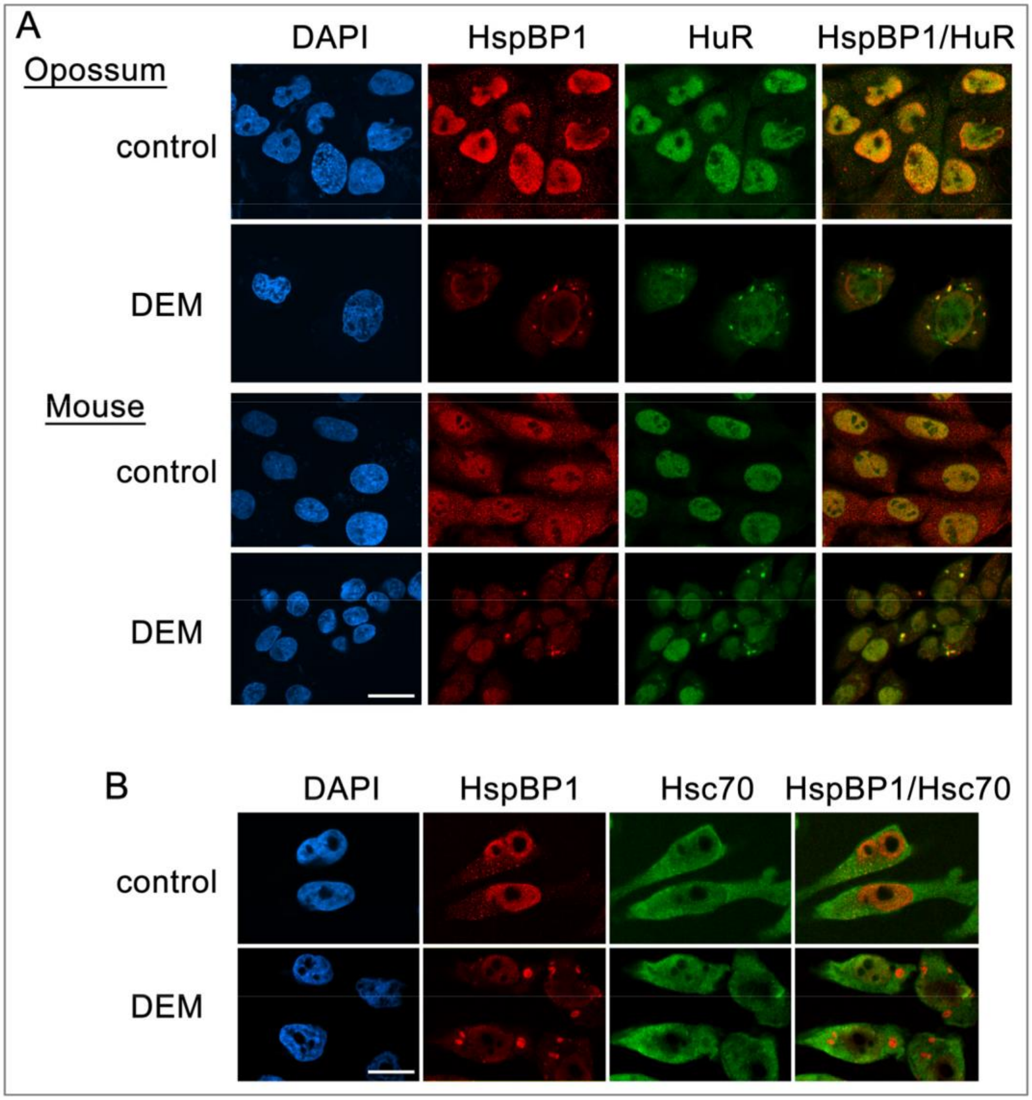

Figure S1. (**A**) HspBP1 associates with stress granules in different mammalian cells. Opossum kidney cells (OK96) and mouse NIH3T3 fibroblasts were incubated with DEM. Using HuR as granule marker HspBP1 was detected in SGs for both cell lines. (**B**) Hsc70 does not concentrate in DEM-SGs. HeLa cells were treated with DEM, followed by immunolocalization of HspBP1 and hsc70. Nuclei were visualized with DAPI, scale bars are 20 µm.

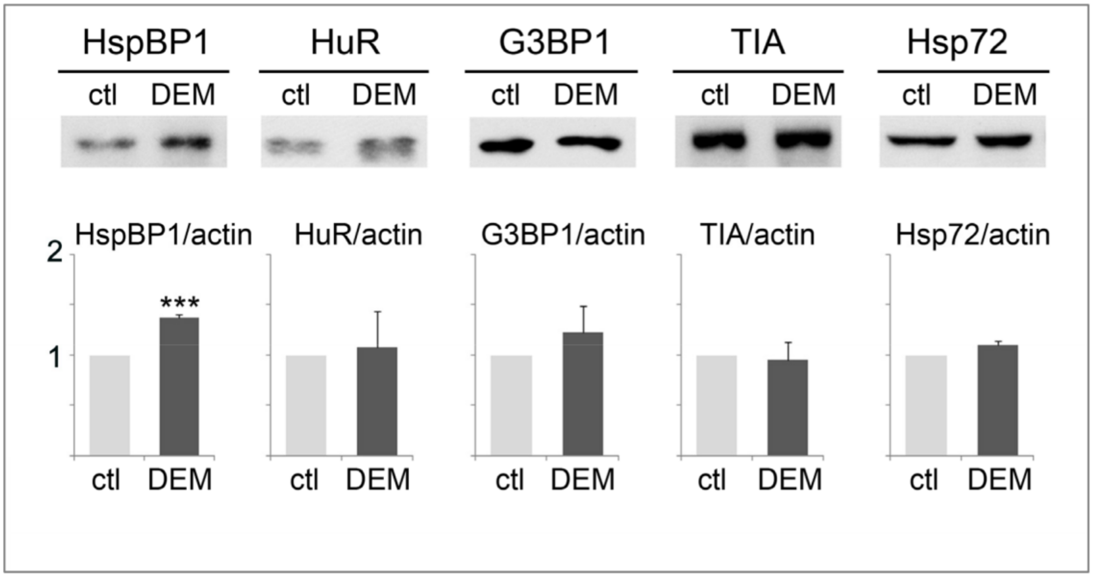

Figure S2. DEM increases the abundance of HspBP1. HeLa cells were treated with vehicle (controls, ctl) or DEM for 4 hours. Crude cell extracts were assessed for the levels of HspBP1, HuR, G3BP1, TIA and hsp72. Changes in protein abundance were determined relative to actin. For each protein, results were normalized to vehicle controls. Data are shown for three independent experiments. Significant differences were identified with Student's *t-test*; *** p<0.001. For each protein, lanes were on the same blot.

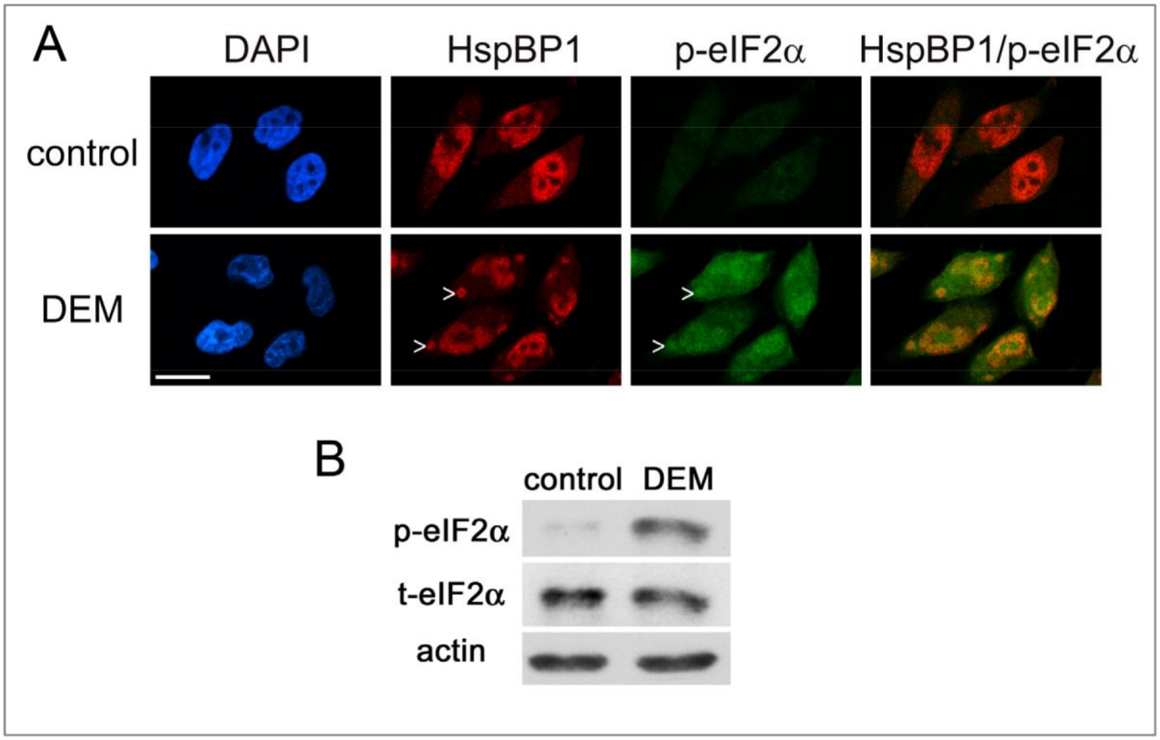

Figure S3. DEM increases the phosphorylation of eIF2α. (**A**) HeLa cells were treated with vehicle (control) or DEM as described for Fig. 1. Antibodies detected eIF2α phosphorylated on Ser51 (p-eIF2α) or HspBP1. Scale bar is 20 µm. The position of two SGs has been marked for the HspBP1 and p-eIF2α panels. (**B**) The phosphorylation of eIF2α and the abundance of total eIF2α (t- eIF2α) were assessed by Western blotting. Actin served as loading control. For each protein examined, the lanes were on the same blot.

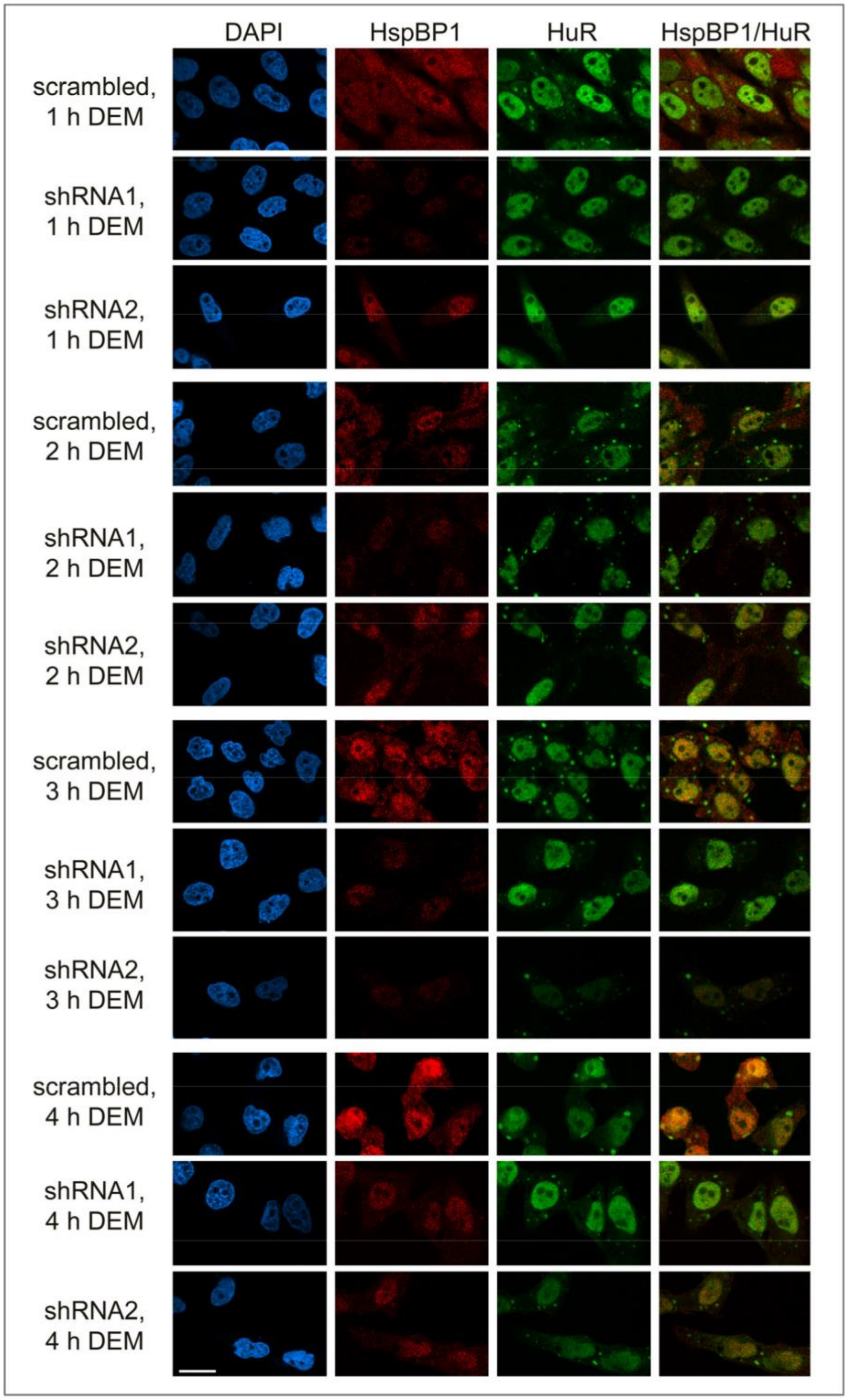

Figure S4. Effect of HspBP1 depletion on the kinetics of SG formation. HspBP1 was depleted with shRNA1 or shRNA2 as described for Fig. 6. Control cells were transfected with a scrambled construct. SG formation was examined after 1, 2, 3 and 4 hours of DEM treatment. HspBP1 and the SG marker HuR were located by indirect immunofluorescence. DAPI demarcated nuclei; scale bar is 20 μm.

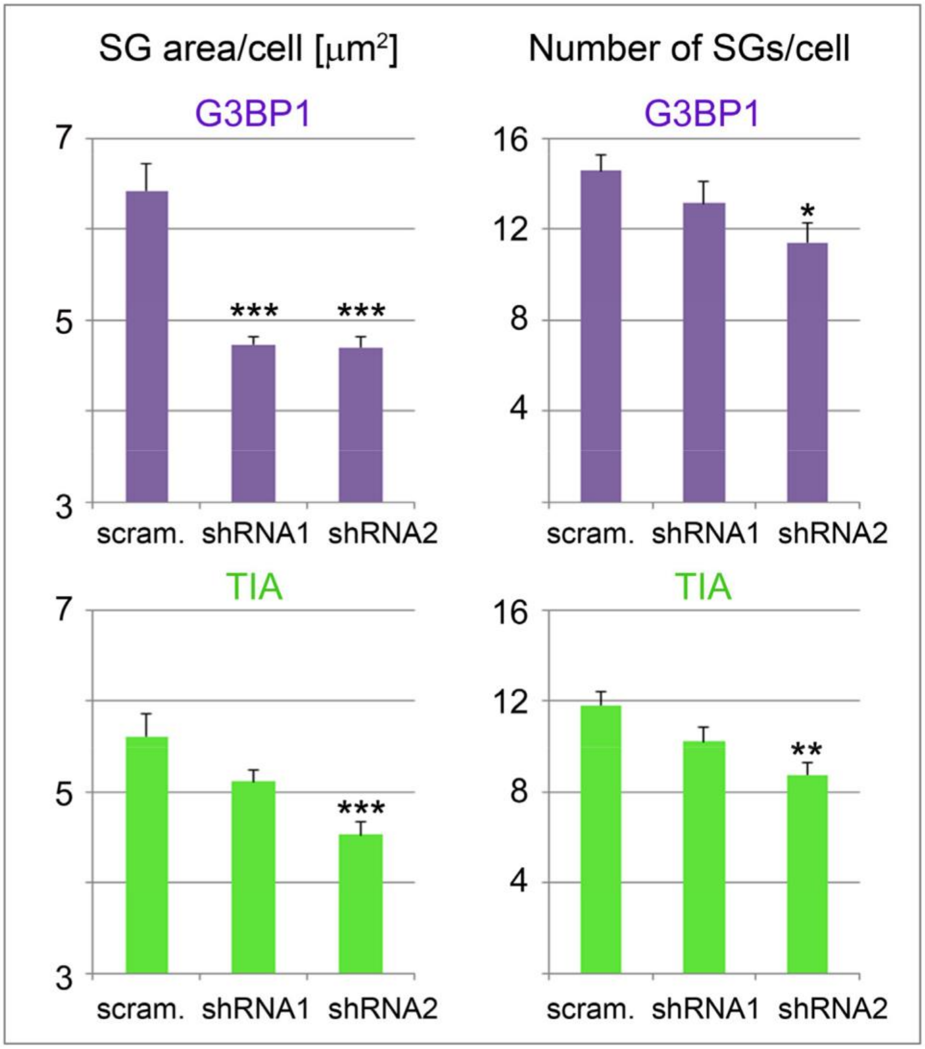

Figure S5. Quantification of granule parameters with G3BP1 and TIA as SG markers. HeLa cells were incubated with plasmids producing scrambled or HSPBP1 silencing RNAs. Cells were treated with DEM, and granule properties were quantified as described for Fig. 4. G3BP1 or TIA was used to demarcate SGs. Statistical assessment was performed with One-Way ANOVA, combined with Bonferroni posthoc analysis; *, $p < 0.05$; **, $p < 0.01$; ***, $p < 0.001$.

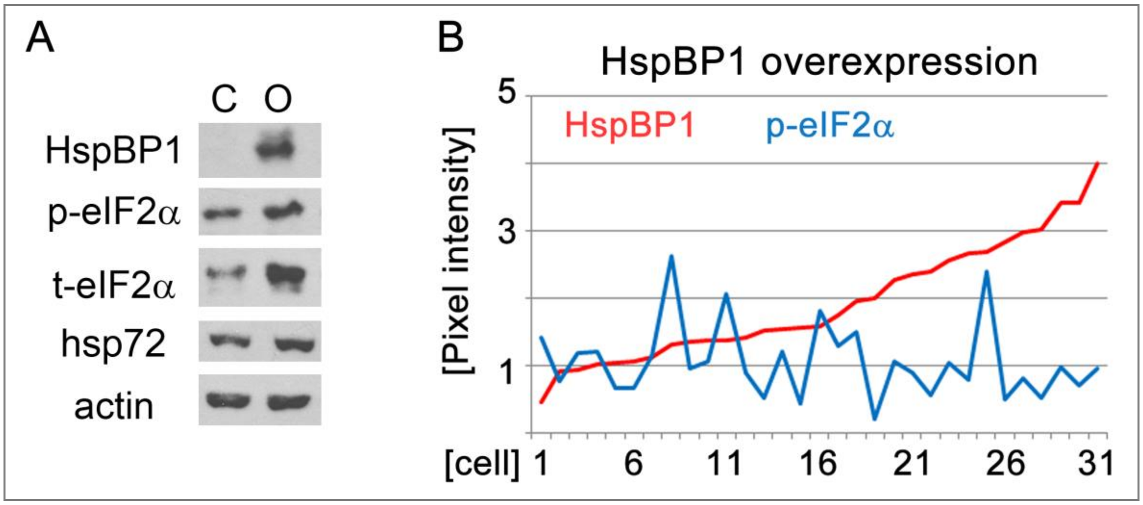

Figure S6. HspBP1 overexpression does not enhance the phosphorylation of eIF2*α*. (**A**) Full length HspBP1 was overproduced (O) in HeLa cells and compared to controls (C). Crude cell extracts were probed with antibodies against HspBP1, eIF2*α* phosphorylated on Ser51 (p-eIF2 *α*), total eIF2*α*, hsp72 and actin. (**B**) Immunostaining combined with quantitative image analysis measured HspBP1 and eIF2*α* phosphorylated on Ser51 (p-eIF2 *α*) for individual cells. Pixel intensities (arbitrary units) were determined at the single-cell level. The signal intensities for HspBP1 and p-eIF2*α* were plotted for individual cells. For each protein, all lanes were on the same blot.

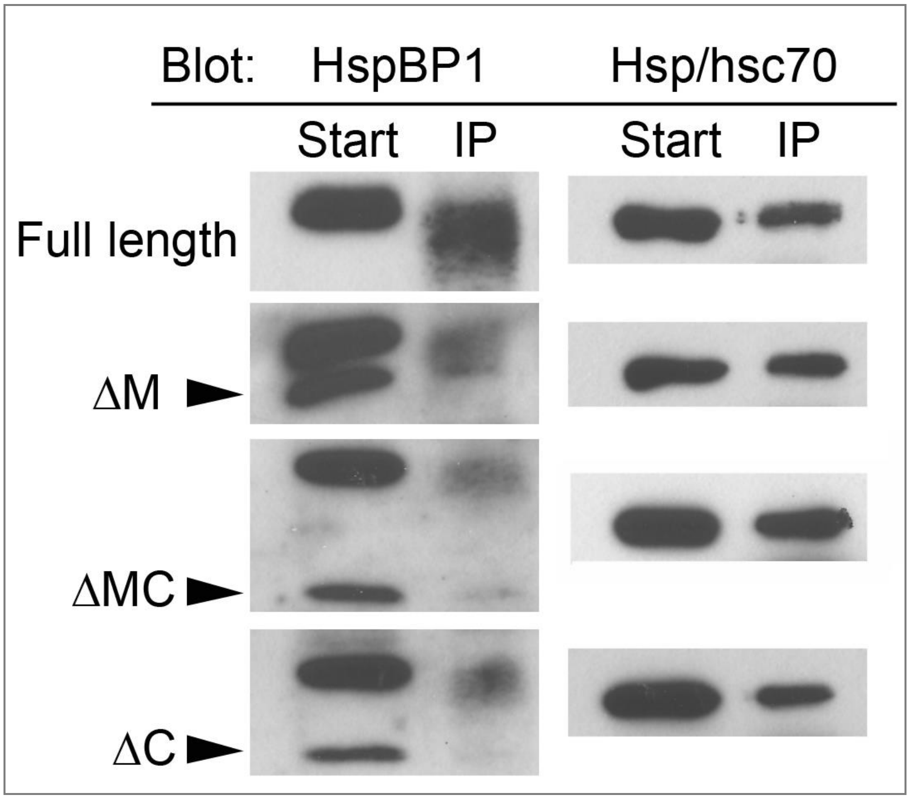

Figure S7. HspBP1 deletion mutants show diminished binding to hsp/hsc70s. HeLa cells were transfected with plasmids encoding full length HspBP1 or the deletion mutants ΔM, ΔMC and ΔC. After transfection, proteins were crosslinked with DSP, and samples were immunoprecipitated with antibodies against hsp/hsc70. Aliquots of the starting material (Start) and immunoprecipitates (IP) were examined by Western blotting with antibodies against HspBP1 and hsp/hsc70. Arrows indicate the position of HspBP1 deletion mutants. For each protein, all lanes were on the same blot.
